# Progress in Drug Delivery to the Central Nervous System by the Prodrug Approach

**DOI:** 10.3390/molecules13051035

**Published:** 2008-05-01

**Authors:** Barbara Pavan, Alessandro Dalpiaz, Nunzia Ciliberti, Carla Biondi, Stefano Manfredini, Silvia Vertuani

**Affiliations:** 1 University of Ferrara, Department of Biology, General Physiology Section, via L. Borsari 46, 44100, Ferrara, Italy; 2 University of Ferrara, Department of Pharmaceutical Sciences, via Fossato di Mortara 19, 44100, Ferrara, Italy

**Keywords:** Brain delivery, nasal administration, prodrugs, SVCT2, carrier-mediated transport

## Abstract

This review describes specific strategies for targeting to the central nervous system (CNS). Systemically administered drugs can reach the brain by crossing one of two physiological barriers resistant to free diffusion of most molecules from blood to CNS: the endothelial blood-brain barrier or the epithelial blood-cerebrospinal fluid barrier. These tissues constitute both transport and enzymatic barriers. The most common strategy for designing effective prodrugs relies on the increase of parent drug lipophilicity. However, increasing lipophilicity without a concomitant increase in rate and selectivity of prodrug bioconversion in the brain will result in failure. In these regards, consideration of the enzymes present in brain tissue and in the barriers is essential for a successful approach. Nasal administration of lipophilic prodrugs can be a promising alternative non-invasive route to improve brain targeting of the parent drugs due to fast absorption and rapid onset of drug action. The carrier-mediated absorption of drugs and prodrugs across epithelial and endothelial barriers is emerging as another novel trend in biotherapeutics. Several specific transporters have been identified in boundary tissues between blood and CNS compartments. Some of them are involved in the active supply of nutrients and have been used to explore prodrug approaches with improved brain delivery. The feasibility of CNS uptake of appropriately designed prodrugs via these transporters is described in detail.

## Introduction

**Figure 1:**
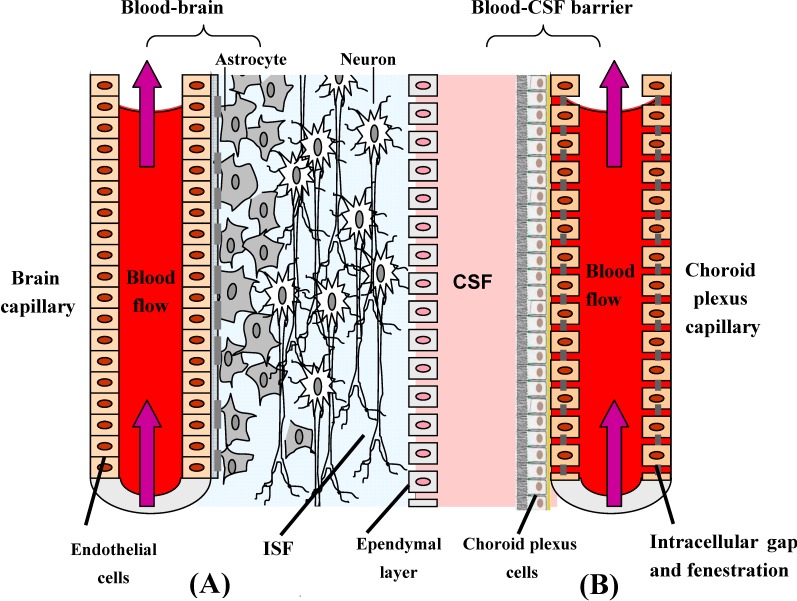
The two main barriers in the CNS: blood-brain barrier (**A**) and blood-cerebrospinal fluid barrier (**B**). ISF: Interstitial Fluid. CSF: Cerebrospinal fluid.

The brain is probably one of the least accessible organs for the delivery of active pharmacological compounds. The same mechanisms that protect the brain from foreign substances also restrict the entry of many potential therapeutic agents. Despite its relatively high blood flow, there are two physiological barriers separating the brain from its blood supply and they control the entry and exit of endogenous and exogenous compounds. One is the blood–brain barrier (BBB) and the other is the blood–cerebrospinal fluid barrier (BCSFB) (see Figure 1).

The BBB is the major barrier to the passage of active molecules from the blood compartment to the brain. The BBB, which segregates the brain interstitial fluid (ISF) from the circulating blood, is located at the level of the brain capillaries, where there is a convergence of different cell types: endothelial cells, pericytes, astrocytes and microglias (perivascular macrophages). The brain microvessel endothelial cells (BMEC) that form the BBB, display important morphological characteristics such as the presence of tight junctions between the cells, the absence of fenestrations and a diminished pinocytic activity, that together help to restrict the passage of compounds from the blood into the extracellular environment of the brain. Tight junctions provide significant transendothelial electrical resistance (TEER) to BMEC and impede the penetration of potential therapeutic agents such as oligonucleotides, antibodies, peptides and proteins [1]. Furthermore, the BMEC express a variety of enzymes, both cytosolic and on the extracellular membrane which also contribute to the restrictive nature of the BBB [2]. Under normal conditions the BBB acts as a barrier to toxic agents and safeguards the integrity of the brain.

The BCSFB separates the blood from the cerebrospinal fluid (CSF) that runs in the subarachnoid space surrounding the brain. This barrier is located at the choroid plexus, and it is formed by epithelial cells held together at their apices by tight junctions, which limit paracellular flux. The CSF-facing surface of the epithelial cells, which secrete CSF into the ventricles, is increased by the presence of microvilli. The capillaries in the choroid plexus allow free movement of molecules via intracellular gaps and fenestrations [3].

Some regions within the CNS lack a BBB and the capillaries are fenestrated allowing the free movement of solutes between the blood and the surrounding interstitial fluid. These areas are collectively termed the circumventricular organs (CVOs) and comprise the choroid plexus, the median eminence, the neurohypophysis, the pineal gland, the organum vasculosum of the lamina terminalis, the subfornical organ, the subcommisural organ and the area postrema. Some of these structures, the median eminence, the neurohypophysis and the pineal are neurohaemal organs specialised for the release of neuroendocrine secretion into the bloodstream. The other areas may be regarded as windows of the brain where a limited number of neurons within the immediate vicinity of the circumventricular organ have an unrestricted access to blood solutes. This access enables the brain to closely monitor the composition of the blood and to react accordingly. The relative surface area of the permeable fenestrated capillaries of CVOs, as compared to the tight BBB capillaries, is 1:5000, so that CVOs do not allow a significant diffusion of substances into the CNS [4,5].

Nevertheless, several disorders and diseases can affect the brain, leading to some loss of BBB integrity. The major neurological diseases affecting the brain may be categorized as neurodegenerative, cerebrovascular, inflammatory (infectious or autoimmune) and cancerous [6]. The diffusion of drugs from the blood into the brain depends mainly upon the ability of the biologically active molecule to cross lipid membranes. Therefore, drugs of interest may not have the requisite physicochemical characteristics necessary to successfully cross the BBB. This is the reason why several strategies have been developed to overcome the BBB [6].

The design of new prodrugs may be a resourceful chemical/biochemical approach to overcome limitations in effectiveness of a parent drug, including solubility. Prodrugs are defined as *per se* therapeutically inactive agents but that can be predictably transformed into active metabolites. In other words, prodrugs act as precursors of parent drugs, with no intrinsic activity, and must undergo, by enzymatic and/or chemical/spontaneous process and in a predictable way, transformation into active agents *in vivo*. Simple prodrugs contain a covalent link between the drug and the strategically selected chemical/transport moiety or promoiety. Thus, the inactive prodrug should ideally release the active agent either non enzimatically at the site of action or by means of target specific enzymes that are either specific for or more abundant at the target site than anywhere else in the body. Obviously, a prodrug must also have easy access to the target tissue [7,8].

## Structure/solubility relationships (prodrugs)

The usual non-invasive approach, to improve the brain drug delivery, is to “lipidize” the drug: the polar functional groups on the drug are masked with non-polar groups, converting a water-soluble drug into a lipophilic “prodrug”.

In actual practice, however, the reformulation of a water-soluble drug by lipidization is a difficult task to accomplish and many different aspects have to be taken into account. Lipinski’s "rule of five" [9], introduced in 1997, is widely used to estimate the solubility and permeability of drugs. Four factors are considered: H-bond donors, H-bond acceptors, molecular weight (MW) and logP.

- An important parameter determining free diffusion of molecules across the BBB is molecular weight. The MWs of virtually all CNS-directed drugs are under 400-500 Da. Lipophilic drugs with masses above the 400-500 Da threshold, with notable exceptions, do not cross the BBB in pharmacologically significant amounts [10]. The biophysical basis for the MW threshold appears to be the transitory formation of pores within the phospholipid bilayers created as the free fatty acyl side-chains kink during the normal molecular motion within the phospholipid bilayers [11]. The pores are of finite size and restrict the movement of small molecules with a spherical volume in excess of the pore volume.
- As a general rule, the BBB permeability of a drug decreases by one log of magnitude for each pair of H-bonds in the form of polar functional group(s), added of the molecule [12]. From its chemical structure it is possible to calculate the number of H-bonds that a given drug forms with water. If their H-bond number does not abide by “Lipinski’s rule of five” it is unlikely that drugs will cross the BBB via lipid-mediated free diffusion in pharmacologically significant amounts. Experimentally the permeation of molecule is more likely when there are up to five H-bond donors (expressed as the sum of OHs and NHs) [13].
- Besides MW and H-bonding, another important factor determining brain availability of a pharmaceutical are the plasma pharmacokinetics and the plasma area under the concentration curve (AUC). The concentration of drug in brain is directly proportional to both the plasma AUC and the BBB permeability coefficient (Pe). Lipidization of the molecule can increase the BBB Pe and also the uptake in all organs of the body, altering the plasma clearance of the drug [14]. The brain uptake of the drug, expressed as percentage of injected dose (ID/g) decreases in proportion to the decrease in plasma AUC caused by lipidization of the drug. Thus, lipidization increases the Pe but decrease the plasma AUC, and these factors can have offsetting effects, resulting in little change in the brain %ID/g.

## Prodrug bioconversion strategies

The fact that a prodrug need conversion into the parent drug to be active makes of outstanding importance the role played by the related biological or chemical processes.

### Esterase activation

A good example of both the advantages of lipidization and a pro-drug approach to CNS delivery is illustrated by the series of related compounds morphine, codeine and heroin [15]. Morphine has a relatively low brain uptake. The classic lipidization approach for this susbtance has involved either the *O*-methylation of morphine to form codeine or *O*-acetylation of morphine to form heroin. As reported above, the BBB permeability to a small molecule increases by a log order of magnitude with the removal of each pair of hydrogen bonds from the parent compound. Therefore, *O*-methylation of morphine, to form codeine, results in the removal of two hydrogen bonds and increases BBB permeability tenfold [15]. The double *O*-acetylation of morphine, to form heroin, increases BBB permeability by approximately 100 fold compared to the parent compound morphine [15]. The ideal lipidization strategy is reversible, and once in the brain, the molecule is enzymatically converted back to the parent compound. Codeine and heroin are converted to morphine in the brain, which interacts with the opioid receptor. Morphine being much more polar than heroin or 6-acetyl-morphine becomes effectively locked into the brain as it cannot diffuse back out across the BBB. This lock-in principle is a major feature of the prodrug approach to CNS delivery.

**Figure 2:**
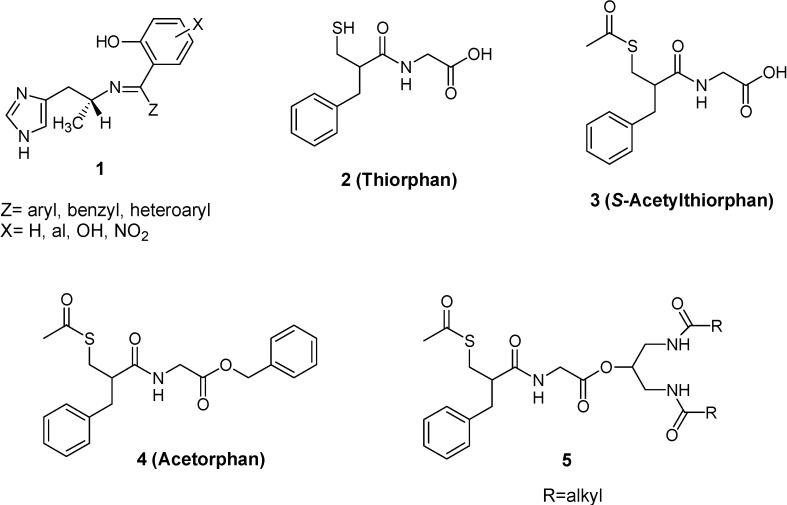
Chemical structures of azomethine prodrugs of (*R*)-α-methylhistamine, thiorphan and prodrugs of thiorphan.

Other examples wherein the prodrug approach coupled to hydrolytic cleavage have been used to solve the BBB drug delivery problem are reported here. Krause *et al.* [16] found that azomethine prodrugs of (*R*)-α-methylhistamine (**1**, a H_3_ receptor agonist having pharmakinetic disadvantages, Figure 2) easily enter the brain through passive diffusion. Indeed, a positive dependence of lipophilicity on brain uptake of this class of prodrugs has been demonstrated. The release of the active drug happens through hydrolytic cleavage of the carbon-nitrogen double bond. This process is essentially chemical and not enzymatically catalyzed.

The delivery of peptides to the brain poses a problem due to their hydrophilic nature and their rapid degradation by peptidases localized within the capillary endothelium [17]. One of the most promising examples of ester linked prodrugs for enhancing the CNS delivery of peptides involves the neutral endopeptidase inhibitor thiorphan, a glycine derivative (**2**, Figure 2). The endopeptidase in this case is a zinc–metallopeptidase, accountable for inactivation of endogenous encephalins, together with aminopeptidase N. The problem of thiorphan is its incapability to cross the BBB [18]. *S*-Acetyl-thiorphan (**3**, Figure 2), the monoacylated form of thiorphan, and acetorphan (**4**, Figure 2), the benzyl ester of *S*-acetylthiorphan, have high analgesic activity, suggesting that their increased lipophilicity improves BBB transport. Following CNS entry they are hydrolyzed by esterase to the more active inhibitor thiorphan [19,20]. It was then demonstrated by Fournie-Zaluski *et al.* [21] that the benzyl ester of acetorphan is rapidly hydrolyzed in serum and so the metabolite *S*-acetyltiorphan accounts for the BBB penetration. Taking advantage of this information Lambert *et al.* [22] have synthesized a series of amide pseudotriglycerides of *S*-acetylthiorphan (**5**, Figure 2) in which the ester bonds in positions 1 and 3 of the glyceride have been replaced by amide bonds in order to increase metabolic stability. These compounds were shown to exhibit analgesic proprieties superior to those of thiorphan and *S*-acetylthiorphan, suggesting that they were acting as prodrugs.

**Figure 3:**
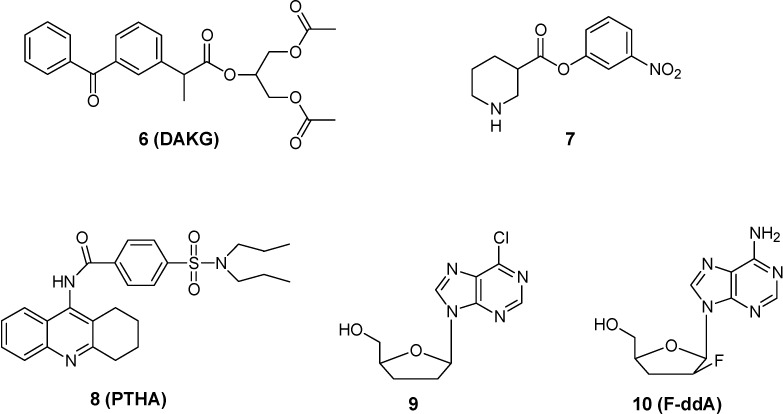
Chemical structures of DAKG, nipecotic acid *m*-nitrophenyl ester, 6-chloro-2’,3’-dideoxypurine, F-ddA and PTHA.

Regarding esterase activation, Yoshiharu *et al.* [23] synthesized a triglyceride prodrug of ketoprofen (1,3-diacetyl-2-ketoprofen glyceride, DAKG, **6**, Figure 3) as a model prodrug for CNS delivery of NSAIDs. The BBB permeability of ketoprofen in the brain-from-plasma direction is very low, because the complete ionization of its carboxyl group at physiological pH and moderate lipophilicity. In addition, the efflux clearance from brain to plasma across the BBB is seven times greater than the influx clearance. Due to these physicochemical and physiological characteristics, the distribution of ketoprofen into the brain is highly restricted. Coupling diacetylglyceride to the carboxylic group of ketoprofen results in an increase of lipophilicity, but this structural modification completely blocks the ionization of the ketoprofen carboxylic acid group. Therefore, it is expected that DAKG can readily cross the brain capillary endothelial cell membrane. Indeed, the study results showed that DAKG improved the delivery of ketoprofen into the brain via increased permeability through the BBB, followed by rapid hydrolysis to ketoprofen within the brain. Although effective, this strategy resulted not useful because the ketoprofen thus generated is readily effluxed from the brain.

The possibility that the process(es) of transformation of drugs into precursors may produce still active molecules instead of prodrugs, once again complicates the interpretation of the observed bioactivities, especially in the absence of detailed pharmacokinetic/pharmacodinamic information. Nipecotic acid, for example, is a potent *in vitro* inhibitor of neuronal and glial uptake of GABA, but it is devoid of *in vivo* activity due to its poor penetration across the BBB [24]. Several prodrug esters of nipecotic acid have been reported, having anticonvulsant activity and effective in inhibiting GABA uptake [25]. Thus, it is unclear whether the anticonvulsive effect is due to the intact ester or to brain tissue esterase activation to nipecotic acid. In fact, Nassereddine-Sebaei *et al.* [26] demonstrated the anticonvulsant effect of the *m*-nitrophenyl ester of nipecotic acid (**7**, Figure 3), which is the most resistant to hydrolysis and the most lipophilic among nipecotic acid prodrugs, providing evidence of a possible effect of the whole molecule. Another example of the usefulness of pharmacokinetic/ pharmacodinamic evaluation of prodrugs is a study by Snead [27], demonstrating that the effects of γ-butyrolactone are the results of its conversation of γ-hydroxybutyrate rather that a direct effect of the lactone itself.

Although ester formation is the most commonly employed approach for increasing lipophilicity of polar molecules exhibiting limited CNS penetration, the unfavourable brain tissue activity of non specific esterases limits the utility of esterase activated prodrugs in enhancing brain/plasma concentration ratios. Efficient targeting via the prodrug approach requires the parent drug to be formed within the target organ at a rate sufficient to compete with its elimination from this latter. To this end, ester prodrugs should be stable to plasma enzymes, but sensitive to those present in brain tissues. This result is difficult to achieve. For example, a series of lipophilic 5'-ester derivatives of 2',3'-dideoxy-inosine (ddI) were evaluated for the improvement of the CNS delivery of ddI [28]. None of the compounds evaluated gave significantly higher CNS concentrations of ddI compared to ddI alone, because esterase activity in plasma far exceeds that in brain tissue, resulting in premature bioconversion of the prodrug. Bulky lipophylic prodrugs might be effective in some cases: niflumic acid was delicered by cholesterol, hexadecanol and by three 1,3-diacylglycerols. The anti-inflammatory activity of these compounds, on experimental brain edema, was evaluated by determination of the prostaglandin E2 (PGE2) brain tissue concentration. Niflumic acid prodrugs with (1,3-dihexadecanoyl-2-[2-[3-(trifluoromethyl)anilino]nicotinoyl] glycerol and 1,3-dihexadecanoyl-2-[2-[3-(trifluoromethyl)- anilino]nicotinoyloxybutanoyl] glycerol showed a marked anti-inflammatory activity at low concentrations [29].

DP-155 is a lipid prodrug of indomethacin that comprises the latter conjugated to lecithin at position sn-2 via a 5-carbon length linker. It is cleaved by phospholipase A2 (PLA)_2_ to a greater extent than similar compounds with linkers of 2, 3 and 4 carbons. Indomethacin is the principal metabolite of DP-155 in rat serum and, after DP-155 oral administration, the half-life of the metabolite was 22 and 93 h in serum and brain, respectively, compared to 10 and 24 h following indomethacin administration. The brain to serum ratio was 3.5 times higher for DP-155 than for indomethacin. The relatively high brain levels of indomethacin after DP-155 administration explain the equal efficacy of DP-155 despite its low systemic blood concentrations [30].

*In vitro* stability studies of oxymethyl-modified coumarinic acid (OMCA) cyclic prodrugs of the diastereomeric opioid peptides DADLE ([D-Ala2,D-Leu5]-Enk, H-Tyr-D-Ala-Gly-Phe-D-Leu-OH), [Ala2,D-Leu5]-Enk (H-Tyr-Ala-Gly-Phe-D-Leu-OH), [D-Ala2,Leu5]-Enk (H-Tyr-D-Ala-Gly-Phe-Leu-OH), and [Ala2,Leu5]-Enk (H-Tyr-Ala-Gly-Phe-Leu-OH) were conducted to evaluate how the chirality of specific amino acid residues (Ala2 and Leu5) in the peptide portion affects their bioconversion by esterases. Comparative studies were conducted in plasma and tissue homogenates (liver and brain) from five animal species (rat, mouse, canine, guinea pig, and hamster) and human. Significant differences in the rates of hydrolysis of the cyclic prodrugs were observed, particularly between cyclic prodrugs with differences in the chirality of the amino acid on the C-terminus of the peptide portion, for example, L-amino acids hydrolyzed more rapidly than D-amino acids. This stereoselective hydrolysis was independent of the animal species, but tended to be more pronounced in brain and liver homogenates, compared to plasma [31]. Prodrug-amenable Thyrotropin-releasing hormone (TRH) analogues were designed by Prokai *et al.* [32] to enhance delivery of a TRH analogue into the CNS; this was achieved through multiple bioreversible lipidation. 3-(Aminocarbonyl)-1-(3-[2-(amino-carbonyl)pyrrolidin-1-yl]-3-oxo-2-{[(5-oxopyrrolidin-2-yl)carbonyl]amino}propyl)-pyridinium showed the high potency and long duration of action in antagonizing pentobarbital-induced narcosis when administered iv in its CNS-permeable prodrug form.

An interesting approach might be the involvement of specific esterases, thus drugs could be converted into ester prodrugs stable to plasma esterase but suitable for degradation induced by specific esterases introduced in the brain by gene-therapy strategies. For example mice with disseminated neuroblastoma tumors have been treated with cDNA encoding a secreted form of rabbit carboxylesterase (rCE). This enzyme activates the prodrug CPT-11 more efficiently than do human enzymes. This neural stem/progenitor cell-directed enzyme prodrug therapy (NDEPT) could be of particular interest in case of targeted therapy for metastatic tumors [33].

Tao *et al.* [34] developed PTHA (9-[P-(*N, N*-dipropylsulfamide)]benzoylamino-1,2,3,4-4H-acridine, **8**, Figure 3) a prodrug of tacrine hydrochloride (THA) prepared by attaching carboxyl group of probenicid to 9-amino group of THA. PTHA is transported into the brain and improves the curative effect of THA, prolongs the activation time and decrease the hepatic toxicity of parent compound. Stable concentration of THA can be kept due to decomposition of the prodrug and gradual release of the parent drug.

### Adenosine deaminase activation

Studies conducted by Singhal *et al.* [35] have confirmed that adenosine deaminase-activated prodrug, such as 6-chloro-2',3'- dideoxypurine (**9**) and F-ddA (**10**) (Figure 3) significantly enhance the CNS delivery of ddI and F-ddI, respectively, due to higher brain tissue activities of adenosine deaminase (ADA) compared with plasma. However, the site of prodrug activation (i.e., the brain microvascular endothelial cells versus the brain parenchymal tissue) is yet to be determined. Thus, it was demonstrated that dideoxynucleoside uptake in the CNS can be enhanced not only through the design of prodrugs that are activated in brain parenchyma, the classical prodrug approach, but also via prodrugs that are activated by enzymes localized within brain microvascular endothelial cells.

### Oxidase activation

Another approach may involve BBB enzymes in the delivery of drugs to CNS. In addition to esterase and adenosine deaminase, a variety of oxidative enzymes, including xanthine oxidase, monoamine oxidase and cytochrome-P450 enzymes, are of particular interest for their role in the enzymatic activity of BBB. These enzymes could be utilized as biotransformation system to in the conversion of drugs unable to cross BBB.

Xanthine oxidase, a constitutive enzyme of all cells involved in purine metabolism, is a metalloflavoprotein which catalyzes the oxidation of a large number of substrates, including hypoxanthine and xanthine. Like ADA, xanthine oxidase is localized in BBB capillary endothelial cells, although its highest activities are found in liver and intestinal mucosa.

Shanmuganathan *et al.* [36] applied the concept of xanthine oxidase-activation to enhanced brain delivery of 2’-F-ara-ddI. 2’-F-ara-ddP prodrug, once evaluated for it CNS delivery potential in mice, was found effective and the study demonstrated that xanthine oxidase was responsible for the metabolism of the prodrug. However, the enzymatic basis of this reaction in the brain has not been elucidated.

Monoamine oxidase (MAO) is a mitochondrial enzyme which catalyzed the oxidative deamination of amines, including a variety of monoamine neurotransmitters.

Milacemide (2-*n*-pentylaminoacetamide) is considered to be a glycine prodrug. It was demonstrated that it readily crosses the BBB and its *N*-dealkylation by monoamine oxidase type B releases glycinamide, which is converted into glycine [37]. Yu *et al.* [38] have recently shown that 2-propyl-1-aminopentane and 2-propylpentylglycinamide are readily deaminated by monoamine oxidase B and by semicarbazide-sensitive amine oxidase. 2-Propyl-1-aminopentane, upon deamination after entry into the brain, leads to valproic acid formation, while 2-propylpentylglycinamide results in the brain tissue formation of both glycine and valproic acid.

## Redox Chemical delivery system

Several other techniques to obtain improved delivery to the brain were developed. Among them, the chemical delivery system (CDS) approach is one of the most interesting procedures for delivering drugs in a sustained and specific manner to the CNS [39]. It involves the release of active species from a lipophilic prodrug through a chemical and/or enzymatic multistep conversion. The CDS approach can be based on a dihydropyridine pyridium salt equilibrium type redox molecular carrier, similar to the endogenous NADH/NAD^+^ coenzyme system. The carrier dihydropyridine is a specific functional group attached to the drug which, in addition to enhancing BBB penetration by virtue of its lipophilicity, can be converted by enzymatic oxidation to a water soluble quaternary pyridinium salt. By intravenous administration, the dihydropyridine form of the CDS form is rapidly distributed throughout the body, including the CNS. Next, the dihydropyridine moiety is oxidized to a membrane-impermeable pyridinium salt. This conversion occurs ubiquitously. The polar, oxidized prodrug (Drug-T^+^) is essentially trapped behind the lipoidal BBB and remains ``locked-in'' in the CNS, while this ionic species formed in the periphery will be rapidly eliminated. The Drug-T^+^ conjugate, which is retained in the CNS, can then undergo to enzymatic hydrolysis releasing the active drug in a slow and sustained manner, whilst the small carrier molecule (pyridimiun salt) is actively transported out of the CNS. Because of the involvement of T ↔ T^+^ redox pairs in this approach of brain targeting, these systems are often called redox CDSs.

**Figure 4:**
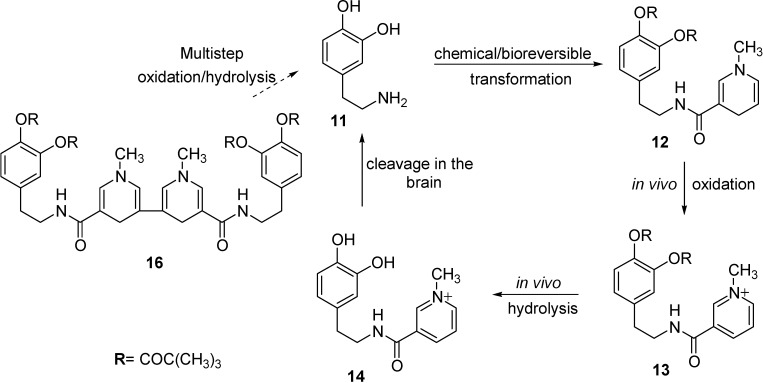
Regeneration of dopamine (**11**) from its amide-type of CDSs (**12** and **16**) in the CNS.

The mechanism of the brain targeting presented in the Figure 4 for dopamine (**11**) can be considered a generic example for the majority of the CDSs. Dipivaloyl-CDS (dopamine-CDS, **12**) was converted to the corresponding quaternary pyridinium salt **13**, followed by sequential esterase-catalyzed hydrolysis to give **14**, the direct precursor of dopamine (**11**).

**Figure 5:**
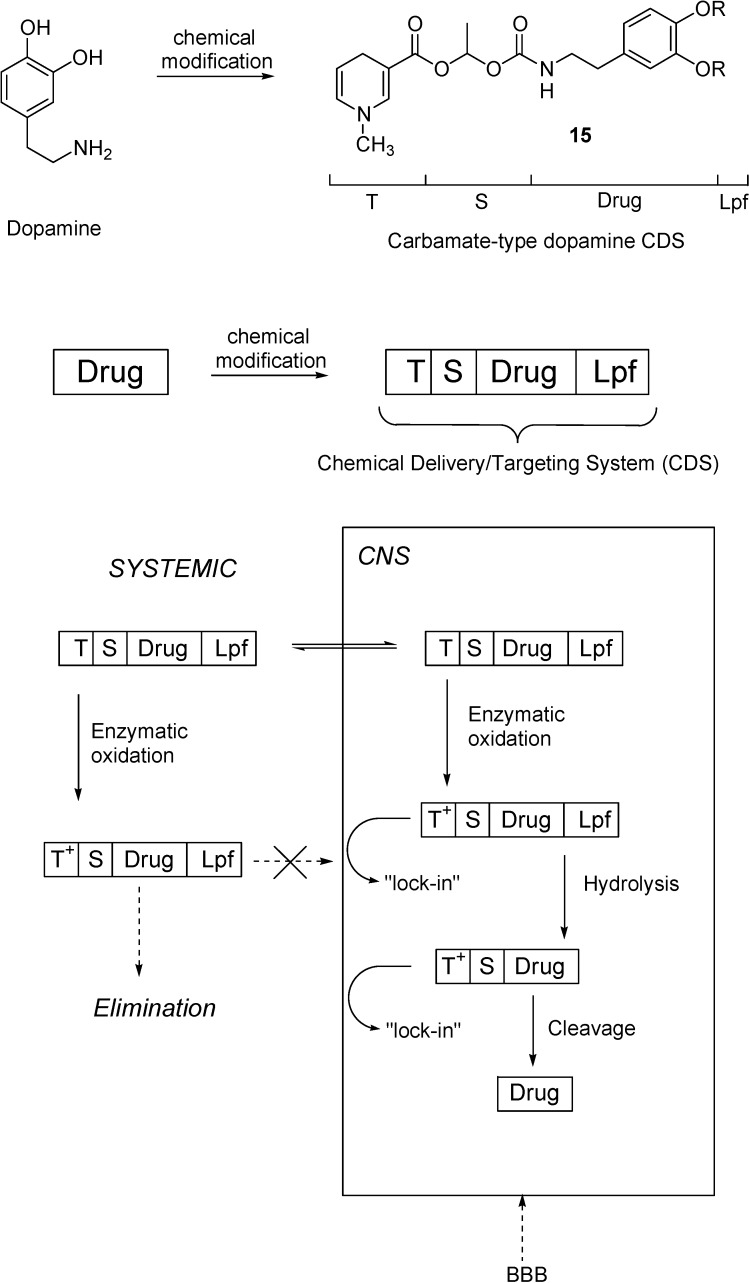
Drug delivery into CNS by molecular packaging and sequential metabolism. The drug is packaged by covalently attached lipophilic groups including a lipoidal **Lpf** and a 1,4-dihydropyridine “targetor” (**T**) that undergoes enzymatic oxidation and turns to a ionic, membrane-impermeable pyridinium moiety (**T^+^**). After distribution in the body and into the CNS by crossing the BBB, the CDS is converted to ionic compounds retained in brain tissue, but ionic conjugates produced in the rest of the body are easily eliminated. The membrane-impermeable conjugates “locked” into the brain undergo sequential metabolism and yield the drug in the CNS. A spacer (**S**) function controls the enzymatic rate of drug release. Scheme was drawn on Prokai [42].

To improve the release of the parent drug hampered by the low amidase-activity in the brain, the T moiety was also attached to **11** through an activated carbamate ester (**15**, Figure 5) [40]. This compound showed a facile conversion to the quaternary pyridinium compound. Recently, a dipivaloyl dimeric CDS (**16**, Figure 4) has also been reported [41].

**Figure 6:**
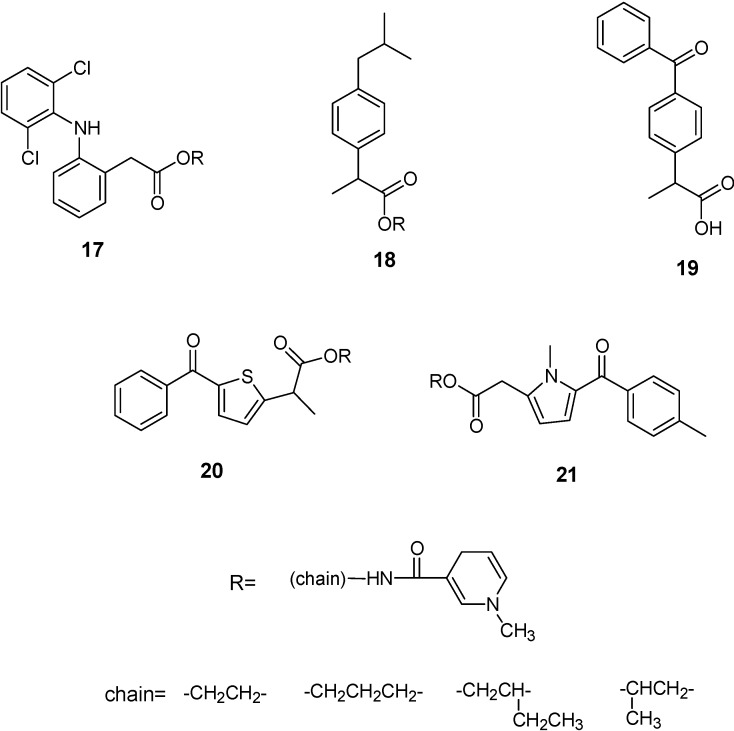
NSAIDs-CDSs.

Perioli *et al.* [43] described some potential prodrugs of several NSAIDs, such as diclofenac, ibuprofen, ketoprofen, tiaprofenic acid and tolmetin (**17**-**21**) (Figure 6) for target drug delivery to the CNS. These compound were synthesized using as a carrier, the 1,4-dihydro-1-methylpyridine-3-carboxylate, which was attached to the drug via an amino alcohol brigde. Predictive BBB penetration studies demonstrated that ibuprofen and diclofenac derivatives were the best candidate for BBB penetration.

**Figure 7:**
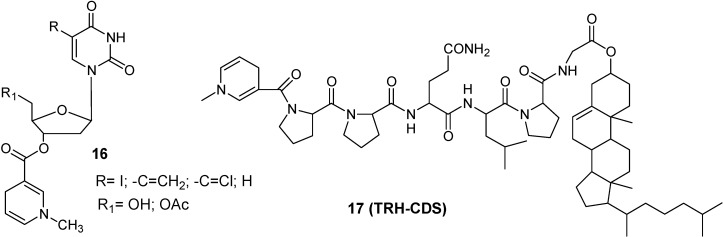
Pyrimidine nucleosides-CDSs and TRH-CDS.

Kumar *et al.* [44] studied 5-substituted pyrimidine nucleosides coupled to a CDS (**22**, Figure 7). They showed that the incorporation of 1-methyl-1,4-dihydropyridyl-3-carbonyl moiety at the C-3’ position of pyrimidine nucleosides significantly increase their lipophilicities, as indicated by the increase in corresponding partition coefficient (P) values, so that they should readily cross the BBB.

The CDS approach has also been extended to achieve successful brain deliveries of enkephalin, TRH and kyotorphin analogues [45,46,47]. The delivery of peptides through the BBB is an even more complex problem than delivery of the other drugs because they can be rapidly inactivated by ubiquitous peptidases. The CDS approach, applied to TRH and shown in Figure 7 (**23**), is a successful delivery system because it solves simultaneously three issues: enhance passive transport by increasing the lipophilicity, assure enzymatic stability to premature degradation and exploit the “lock-in” mechanism to provide targeting [48,49].

## Transport processes in the CNS: prodrugs acting through carrier-mediated BBB crossing

In the absence of lipid-mediated mechanism, circulating molecules can penetrate into the central nervous system (CNS) by interaction with endogenous transport systems located within the brain capillary endothelium or the neuroepithelial cells of the choroid plexus. The endogenous transporters can be classified into three main categories [50]:

(a) CMT (carrier mediate transport), suited to generally transport from blood to CNS compounds with a molecular mass smaller than 600 Da. These systems can be concentrative, with energy employment (ATP-driven or Na^+^-dependent), or equilibrative (facilitate transport) without energy employment [51]. Several prodrugs have been designed and synthesized to interact towards CMT systems in the aim to obtain the uptake of drugs into the central nervous systems. A description of these strategies will be reported below.
(b) AET (active efflux transport), able to expel a multiplicity of drugs from the CNS to the blood-stream. As a consequence, some drugs cannot penetrate into the brain, being substrates for BBB AET systems that have been identified responsible for the occurrence of multidrug resistance. The development of co-drugs that inhibit the AET systems can be a strategy for increasing brain penetration of drugs [52].
(c) RMT (receptor mediated transport), able to internalise relatively large compounds (peptide and proteins) via an endocytotic process. These systems are studied for targeted delivery to the brain of drugs with high molecular weight [51].

## Prodrugs and Carrier Mediated Transport (CMT)

CMT systems transport nutrients, vitamins or hormones into the central nervous system. This type of transport was firstly investigated *in vivo* with physiologic techniques [53,54] allowing determination of the Michaelis-Menten kinetic parameters (K_m_, expressing the substrate-transporter affinity and V_max_, expressing the transporter capacity) [54,55]. More recently, the progress of molecular cloning of transporter genes and their expression in cultured cells, have consented to greatly extend knowledge on membrane transport mechanisms [56] to understand how the transporters can be employed for the brain targeting of drugs. Table 1 reports CMT systems for six classes of nutrients transported into the central nervous system. The genes of these transport systems have been identified [52,56,57]. The transporters of neutral amino acids (LAT 1), hexose (GLUT 1) monocarboxylic acids (MCT 1), cationic aminoacids (CAT 1) and nucleosides (CNT2) are widely expressed at the BBB level, whereas the ascorbic acid transporter (SVCT2) is mainly expressed in the choroids plexus.

In general, CMT systems are highly stereospecific for their substrates or, in other words, they display significant structural requirements. As a consequence, neuroactive drugs themselves are not transported by CMT systems, but pro-drugs approaches have been proposed to overcome these drawbacks. They are based on two main different strategies: (i) the modification of drug into a “pseudonutrient” structure, able to be transported by a CMT system; (ii) the drug conjugation with a nutrient able to be CMT transported. In both cases, the drugs are released after enzymatic cleavage from their prodrugs after being targeted into the central nervous system. These approaches have been developed for the carriers LAT 1, GLUT 1 and SVCT 2, here following some examples.

**Table 1:** CMT systems involved in the transport of nutrients into the central nervous system.

Carrier	Type	Representative substrate	Main expression in blood/CNS barriers
Neutral amino acid	LAT 1	Phenylalanine	Blood-brain barrier
Hexose	GLUT 1	Glucose	Blood-brain-barrier
Monocarboxylic acid	MCT 1	Lactic acid	Blood-brain-barrier
Cationic amino acid	CAT 1	Arginine	Blood-brain barrier
Nucleoside	CNT 2	Adenosine	Blood-brain barrier
Ascorbic acid	SVCT 2	Vitamin C	Choroid Plexus

### Prodrugs and LAT 1 system

The transporters of aminoacids are classified in terms of their sodium dependence (functional characteristic) and their substrate specificity. LAT subtypes are sodium-ion-independent transporters of large neutral aminoacids (as an example, phenylalanine, tyrosine or leucine) and are expressed in the BBB [56]. The first cloned gene of the system L transporter has been named LAT 1 and detected in the brain by Northern blot analysis [58]. Transporters LAT 2, whose genes were more recently cloned, have a different substrate specificity from LAT 1 [59,60]. Currently, the principal large neutral amino acid transporter at the BBB is identified as the LAT 1 [54].

**Figure 8:**
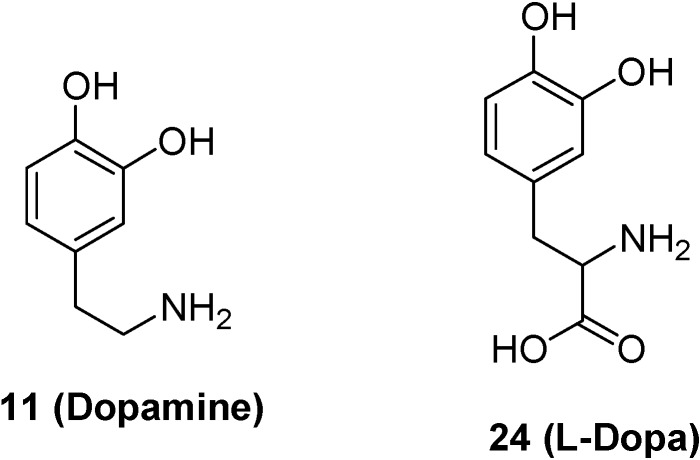
Chemical structures of dopamine and its prodrug l-DOPA as “pseudonutrient” for LAT 1 system.

The use of l-DOPA (**24**, Figure 8) for the brain targeting of dopamine, which does not cross the BBB, is a classical example of modification of the drug structure, with the aim of obtaining a prodrug as a “pseudonutrient” substrate for LAT 1 [52]. Indeed, the α-carboxylation of dopamine allows one to obtain l-DOPA that, being a large neutral aminoacid, is a good substrate for LAT 1 (Fig. 8). The aromatic amino acid decarboxylase induces the decarboxylation of l-DOPA, which is transported into the central nervous system, and, therefore, the dopamine delivery into the brain. The supplementation of this drug alleviates the neurological symptoms associated with Parkinson’s disease and, currently, the usual treatment for this disease is L-DOPA [61].

**Figure 9:**
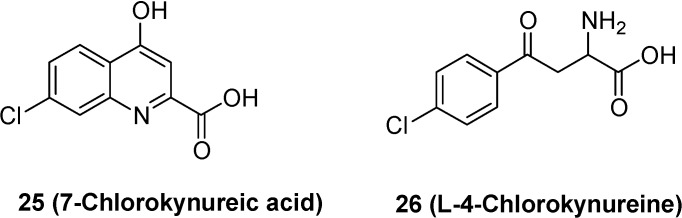
Chemical structures of 7-chlorokynurenic acid and its prodrug l-4-chloro-kynureine as “pseudonutrient” for LAT 1 system.

Another example is represented by l-4-chlorokynureine (**25**) that can be considered a prodrug of 7-chlorokynurenic acid (**26**, Figure 9) [62]. This drug is an antagonist of NMDA receptors and, therefore, it can be an effective as neuroprotective agents. On the other hand, its low lipid solubility do not allows the penetration in the brain, at therapeutic concentrations, after systemic administration [63]. It has been demonstrated that l-4-chlorokynureine, being a large neutral aminoacid, is a substrate of LAT 1, and that kynurenine aminotransferase [64] provides intracerebral conversion of the prodrug to chlorokynurenic acid [62].

**Figure 10:**
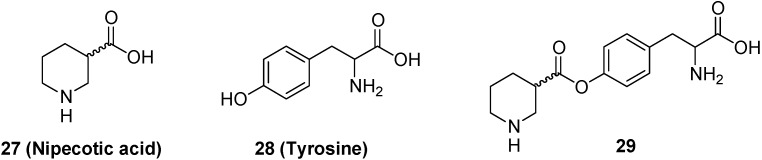
Chemical structures of nipecotic acid, tyrosine and their ester. Nipecotic acid shows anticonvulsant activity only when administered as the ester.

Prodrugs transported by LAT 1 were also obtained by means of the conjugation of neuroactive drugs with neutral amino acids. For example, a prodrug **29** (Figure 10) of nipecotic acid (**27**) was obtained by conjugation with tyrosine [65].

Nipecotic acid is a potent inhibitor of neuronal GABA uptake, and therefore it can be an effective anticonvulsant [66]. As for kynurenic acid, it is inactive when administer systemically, being unable to reach the brain from the bloodstream [67]. It has been demonstrated that intraperitoneally injected nipecotic-tyrosine ester is able to protect mice against audiogenic seizures in a dose dependent manner, suggesting that the prodrug is transported into the brain by an amino acid transport system [65].

**Figure 11:**
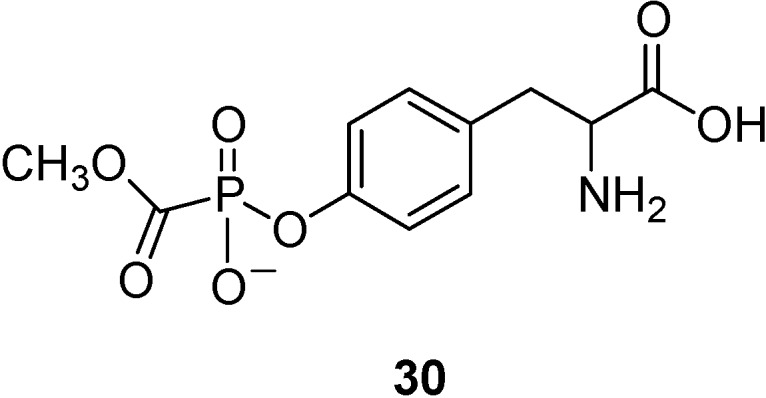
Chemical structure of the phosphonoformate-l-tyrosine conjugate.

A similar example is constituted by the conjugation of phosphonoformate, an antiviral agent [68], with l-tyrosine (**30**, Figure 11) [69,70]. It has been demonstrated by *in vitro* studies, that the tyrosine prodrug of phosphonoformate is a good substrate of LAT 1 expressed in monolayers of porcine brain microvessel endothelial cells [69].

### Prodrugs and GLUT 1 system.

d-Glucose is an essential nutrient for the brain functions, able to penetrate the CNS via appropriate transporters. At the BBB, the capacity of glucose transporters is about 15 and 50 times higher than those of monocarboxylic acid and neutral aminoacid transporters, respectively [56]. Glucose transporters can be classified into two main families: sodium dependent (secondary active, SGLT) and sodium independent (facilitative, GLUT). There are over a half-dozen members of the GLUT transporter gene family, but GLUT 1 constitutes over than the 90% of BBB glucose transporters [54,71]. GLUT 1 transports d-Glucose, 2-deoxyglucose, 3-*O*-methylglucose, but not l-glucose [72]. 3-*O*-Methylglucose is non-metabolizable and, therefore, its radiolabelled analogue ([^3^H]3-O-methylglucose) was used as tracer to characterize glucose transporter mediated processes [73].

Conjugation of drugs with d-Glucose has been proposed as a strategy to improve their uptake into the brain. As an example, it has been demonstrated that the conjugation of the β-d-Glucose moiety to opioid agonist peptides decreased their lipophilicity, but paradoxically, increased their transfer across the BBB of rats [74]. Moreover, glycopeptides administered to mice appeared more potent analgesic than the unglycosilated peptides [75,76]. It was hypothesised a role of GLUT 1 transporter for the brain uptake of the glycosilated peptides [75], even if additional studies are needed to evaluate this aspect, this observation is intriguing [56].

**Figure 12:**
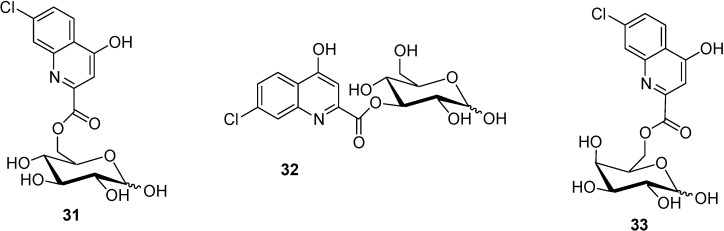
Chemical structures of the glycosyl-conjugates of 7-chlorokinurenic acid (7-Cl-Kyn). The 7-Cl-kyn-glucopyranos-6'-ylester was more potent as anticonvulsant in mice than the 7-Cl-kyn-glucopyranos-3'-ylester. The 7-Cl-kyn-galactopyranos-6'-ylester was not protective in mice against NMDA induced seizures.

Similarly, after intraperitoneal administration the ester conjugation of 7-chlorokynurenic acid with d-Glucose induced protective effects in mice against seizures induced by NMDA [77,78]. In particular, it was found that d-glucopyranos-6’-yl ester **31** was more potent than d-glucopyranos-3’-yl ester **32** (Figure 12), whereas, the conjugation with galactose (galactopyranos-6’-yl ester **33**) was not protective against NMDA seizures. According to these results, a role of GLUT 1 was hypothesized for the brain uptake of the two active compounds, taking into account that this transporter has a lower affinity for d-galactose than for d-Glucose [79]. These studies have also evidenced that the substituted position of d-Glucose is important for absorption and metabolism of d-Glucose conjugates [77,78].

**Figure 13:**
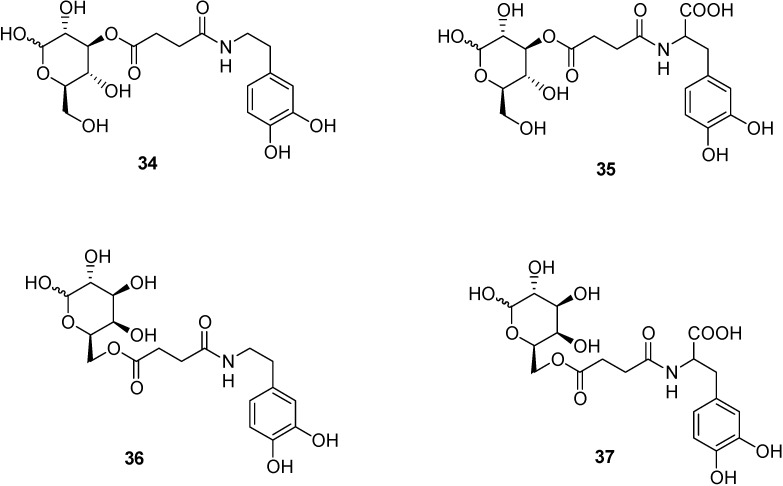
Chemical structures of the glycosyl conjugates of dopamine and l-DOPA. All compounds were able to induce in rats therapeutic effects against Parkinson’s disease, after intravenous administration. The dopamine-glucose conjugate was demonstrated to be transported by the human GLUT 1 transporter expressed by human retinal pigment epithelium (HRPE) cells.

On these bases, glycosyl conjugates of dopamine and l-DOPA were obtained, via a succinyl linker, as esters at C-3 position of glucose (**34**, **35**) and at C-6 of galactose, (**36**, **37**, Figure 13). In this case, dopamine derivatives appeared more active in reversing reserpine-induced hypolocomotion in rats, than l-DOPA or its esters. All compounds were able to reduce morphine-induced locomotion [80].

These results lead to hypothesize that the glycosyl conjugation of dopamine consented the crossing the BBB of the drug by GLUT 1 interaction and transport.

Very recently we have demonstrated that, unlike dopamine, the dopamine-glucose conjugate (**34**, Figure 13) is a transportable substrate of GLUT 1 transporter expressed by human retinal pigment epithelium (HRPE) cells. Indeed, dopamine was able to inhibit GLUT 1 mediated uptake of [^3^H]3-*O*-methylglucose into the cells only if conjugated to glucose. Moreover, the uptake of the conjugate into HRPE cells was inhibited by glucose, confirming the aptitude of dopamine glycosyl ester to be a transportable substrate of glucose transporter [81].

### Prodrugs and the SVCT 2 system.

**Figure 14:**
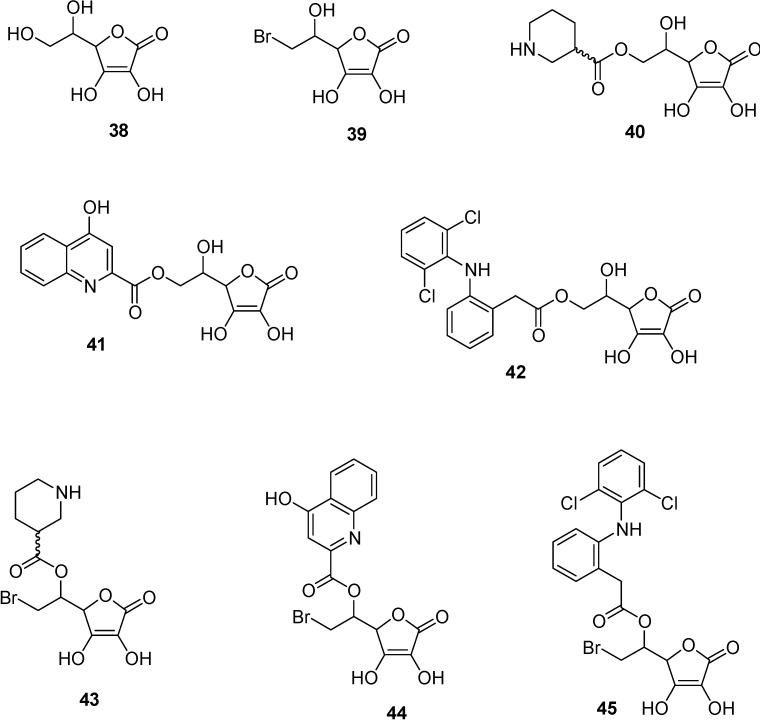
Chemical structures of vitamin C, its Br-ascorbate and their conjugate with nipecotic acid (Nipec), kynurenic acid (Kynur) and diclophenamic acid (Diclo).

Vitamin C (ascorbic acid, **38**, Figure 14) is not synthesized by humans [82], despite the fact that this nutrient is essential for eyes, spinal cord and brain of all mammals. In particular, vitamin C is necessary as a neuromodulator, for acetylcholine and noradrenaline release, or it contributes to myelin formation [83,84,85,57]. In the cerebrospinal fluid (CSF) the concentration of vitamin C is about 500 μM, tenfold higher than the concentration generally found in the plasma of mammals. Moreover, the vitamin C concentration is estimated about 10 mM in neurons and 1 mM in glia [57]. The intake of vitamin C in the body compartments of mammals is guaranteed by a class of Na^+^ dependent transporters recently characterized and called SVCT [86,87,88,89]. In particular, SVCT1 allows the absorption of vitamin C from the intestine and its recovery by the kidneys; SVCT2, instead, allows accumulation of the vitamin in the brain and eye, being expressed by neuroepithelial cells of the choroid plexus and the retinal pigment epithelium, respectively [84,85,57]. According to these aspects, SVCT2 was considered by us as a potential candidate for the transport of neuroactive drugs into the CNS. As a consequence, we have investigated the conjugation with vitamin C as a possible tool to obtain the CNS uptake of neuroactive drugs that are not effectively delivered into the brain [90]. Nipecotic, kynurenic and diclophenamic acids were chosen as model compounds, being potentially able to induce therapeutic effects against important CNS pathologies - such as epilepsy, neuronal disorders, Parkinson’s and Alzheimer’s diseases - but unable to reach the brain from the bloodstream [90,91]. Their conjugates **40**-**45** with vitamin C are shown in Figure 14. During this study we have demonstrated that human retinal pigment epithelium (HRPE) cells selectively express the SVCT2 transporter [90]. We have therefore chosen this cell line [92] to perform *in vitro* analysis about the interaction of the drugs and their conjugates. It was demonstrated that nipecotic and kynurenic acids can interact toward SVCT2 only as ascorbate conjugates. In this case they appeared as competitive inhibitors of the vitamin C transport mediated by SVCT2. A similar behaviour was found with their Br-ascorbate conjugates (**39**, Figure 14), whose affinity toward SVCT2 appeared increased, in comparison with the ascorbate conjugates [93,94]. These results indicated that, after conjugation nipecotic and kynurenic acids, were to interact toward SVCT2 in the same binding site of vitamin C.

Differently from nipecotic and kynurenic acids, diclofenamic acid was able to interact towards SVCT2 as a non competitive inhibitor and its affinity appeared about tenfold higher than that of the endogenous vitamin [90,95,96]. The conjugation with vitamin C allowed further improvement of the affinity of this drug towards SVCT2, but changed its behaviour from a non competitive to a competitive inhibitor [90]. On the other hand, the conjugation with Br-ascorbate induced an affinity decrease [93,94].

Further investigations demonstrated that the nipecotic acid conjugate with Br-ascorbate was transported into HRPE cells by SVCT2, whereas the kynurenic acid conjugate was not accumulated into the cells [93]. This difference can be attributed to the different steric hindrance of the two conjugates, and suggests that SVCT2 is highly selective for the transport of its substrates.

To support our *in vitro* data we evaluated the effects on pentylenetetrazol-induced convulsions in mice of systemic injection of of nipecotic and kynurenic acids and their conjugates: only the conjugates of nipecotic acids were able to induce anticonvulsant effects. Moroever these effects were inhibited in the presence of dicofenamic acid, that was found to be a non competitive inhibitor of SVCT2 mediated transport of vitamin C [94,95,96].

## Prodrugs and carriers: a valuable strategy?

In general, the carriers of nutrients are very selective in their structural needs for substrates. As an example, it has been reported that the hexose transporter GLUT1 is very stringent in its stereochemical requirements for transport [5]. As discussed above, it has been hypothesized that a drug which is not transported into the brain, may become transportable by its conjugation with endogenous CMT substrates. It has been also proposed that it might be better to modify the structure of a pharmaceutical into a pseudonutrient [52]. To this purposes, l-DOPA is the first example of a pro-drug that crosses biological membranes, via carrier mediated transport. The analysis of data above reported suggests that both these strategies may be useful or not depending on the transporter chosen. Indeed, the LAT1 system appears rather versatile, being able to transport either pseudonutrients or drug conjugates [97]. The GLUT 1 system appears highly selective towards the transport of molecules different from d-Glucose, but it seems promising for the transport of drugs conjugates with this nutrient.

Very high selectivity is shown by SVCT2 system, not particularly for the recognition of substrates, but for their transport. In this case the conjugation mediated transport appears allowed for very small molecules. The studies performed on SVCT2 and GLUT 1 systems shows that the expression of the transporters in cellular lines can allow to obtain detailed information about the molecular mechanisms that regulate the transporter-substrate interactions. Similar studies may be therefore of great utility for the design and synthesis of prodrug able to be CMT transported.

## Trojan horses

Endogenous large-molecule peptides, such as insulin, transferrin, and leptin are transported across the BBB via the receptor-mediated transcytosis (RMT) process, which operates in parallel with the classic CMT systems. The insulin receptor or the transferrin receptor (TfR) are expressed on the plasma membrane of the brain capillary endothelial cell and serves to transport endogenous insulin or transferrin from blood to brain. Similarly, certain peptidomimetic monoclonal antibodies (MAb) undergo RMT across the BBB by the endogenous peptide receptor transporters. The peptidomimetic MAbs bind exofacial epitopes on the BBB RMT system, which triggers transport across the BBB. Since the MAb binding site is different from the binding site of the endogenous ligand, there is no interference of endogenous ligand transport. The peptidomimetic MAbs may be used as molecular Trojan horses (mAb THs) to ferry large therapeutic molecules, including protein, antisense nucleotide, or non-viral plasmid DNA across the BBB via the endogenous RMT systems. Molecular Trojan horses provide a brain drug targeting technology that allows the non-invasive delivery of large molecule therapeutics to the human brain [98]. A board of receptor-specific mAb THs has been developed for BBB drug delivery in rodents, primates and humans. mAb-based THs are species specific. For drug delivery in mice, the rat 8D3 mAb, which recognizes the mouse transferrin receptor (TfR), is used; whereas for drug delivery in rats, the murine OX26 mAb, targeting the rat TfR, is used. For drug delivery to Old World primates such as the rhesus monkey, the murine 83-14 mAb, targeting the human insulin receptor (HIR), is used, and for drug and gene delivery to humans, genetically engineered forms of the HIR mAb are employed [98]. Non-antibody delivery systems have been also developed, including histones, p97, receptor-associated protein, the Tat transduction domain peptide, and other cationic peptides or polymers. Although the transport of ligands is hypothesised to be receptor-mediated, the transport of cationic peptides might be mediated on the basis of charge interactions by absorptive-mediated endocytosis systems: the conjugation of low-density lipoprotein apoproteins to the surface of nanoparticles triggers receptor-mediated transcytosis across the BBB by the low-density lipoprotein receptor in the BBB [99].

Preclinical studies in rodents or primates show that the attachment of the drug to TH is facilitated by the use of avidin–biotin technology. In this approach, the drug is mono-biotinylated and a conjugate of the targeting mAb and avidin or streptavidin (SA) are produced. Owing to the very high-affinity binding of biotin to avidin or SA, there is instantaneous attachment of the biotinylated drug to the targeting mAb. The vial containing the biotinylated drug is mixed with the vial containing the TH–SA conjugate just before intravenous administration in animals. The complex of a nontransportable drug (D) and TH is called chimeric peptide because the molecule is bifunctional. The TH part of the molecule binds a specific receptor, R_1_, on the BBB to enable the transport into brain. The D part of the molecule binds its cognate receptor, R_2_, on brain cells to initiate the pharmaceutical effect, once the chimeric peptide penetrates into brain [98].

## Nasal administration for the brain delivery of drugs

Recently, the nasal route of administration has gained increasing consideration for obtaining systemic absorption or brain uptake of drugs [100,101], due to the high vascularization of the nasal mucosa [102]. In the nasal cavity the respiratory region has the highest degree of vascularity. The olfactory region is located in the top of the nasal cavity and it is the only site of the body where the CNS is in contact with the external environment.

By this way drugs can be absorbed into the blood stream across the nasal membrane of the respiratory region. Lipophilic molecules are easily transported via a transcellular mechanism. On the other hand, polar or hydrophilic molecules pass the nasal membrane via a paracellular mechanism that is dramatically less efficient than the transcellular pathway. As a consequence, polar or hydrophilic molecules may have some chances to reach the olfactory mucosa and to be transported into the CNS. Indeed, the nerves belonging to the olfactory epithelium are surrounded by CSF in a space that is continuous with the subarachnoidal one: after intranasal administration the drugs able to reach the olfactory region can, therefore, diffuse into the cerebrospinal compartment through the olfactory epithelium via transcellular or paracellular mechanisms [100,101,102]. The drugs can then diffuse into the interstitial fluid (ISF) from where it can penetrate the brain parenchyma [103,104]. As an example, nasal administration of dopamine solutions allows to obtain uptake into the brain of this neuroactive drug [105]. Drugs can also be transported into the brain by trigeminal nerves that reach the nasal cavity [103,104,105,106].

The nasal mucosa includes not only a physical barrier constituted by mucus and epithelium, but also a temporal barrier controlled by the mucociliary clearance and an enzymatic barrier including aldehyde dehydrogenase, glutathione transferase, epoxide hydrolase, carboxylesterases and aminopeptidase [102]. This enzymatic activity can create a “pseudo” first pass effect for drugs nasally administered. The nasal route can be also constrained by a not adequate aqueous solubility of drugs, because the entire dose is given in a volume o 25 – 200 μL, requiring, thus, high aqueous solubility.

## Brain targeted nasal prodrug delivery

A prodrug approach can be very useful to change the chemical-physical properties of nasally administered drugs in the aim to enhance their ability to cross the nasal mucosa and to prevent their metabolic degradation. The linkage of a biocleavable moieties to drugs can induce significant increase of their bioavailability. As an example, the synthesis of ester prodrugs allows to easily introduce different functionalities at hydroxyl or carboxylic groups of drugs, changing their octanol/water partition coefficient and their stability in physiological fluids. Moreover, chemical or enzymatic bioconversion can be controlled by appropriate substitutions [102,107].

**Figure 15:**
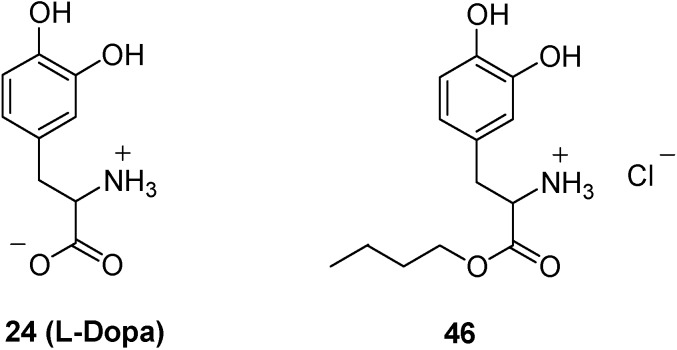
Chemical structures of l-DOPA and its ester prodrug.

The importance of nasally administered prodrugs can be examplified as follow. Less than 1% of orally l-DOPA administered reaches the brain unchanged, being transformed to dopamine during first-pass metabolism [108]. Dopamine induces, at peripheral, side effects such as nausea, vomiting and cardiac irregularities [108]. l-DOPA is therefore a good candidate for nasal administrations, but, unfortunately, this drug is not very soluble. In the aim to improve the efficacy of liquid nasal formulations (> 10 mg in 100 μL), l-DOPA has been transformed in its butyl ester, giving a pro-drug more lipophilic and water soluble than the parent compound. The DOPA structure has been indeed changed from a zwitterionic form to an amine salt (**46**, Figure 15). In rat *in vivo* studies substantiated that after nasal administration, this prodrug resulted rapidly adsorbed into the systemic circulation and converted to l-DOPA without significant formation of dopamine. Moreover, an improvement of l-DOPA adsorption into CNS was detected in comparison with equivalent intravenous doses [109]. It would appear that the utilization of water soluble prodrugs of l-DOPA via the nasal route may have therapeutic success in the treatment of Parkinson’s disease [109].

**Figure 16:**
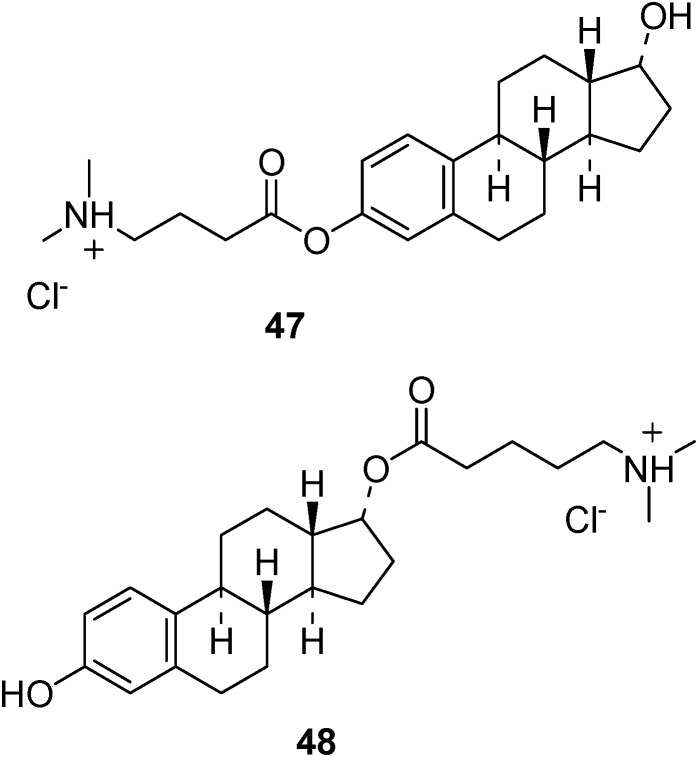
Chemical structures of 17β-estradiol ester prodrugs.

Similar results were obtained with ester prodrugs of 17β-estradiol (**47**, **48**, Figure 16), a drug that can decrease the risk of Alzheimer’s disease, but that show poor water solubility for nasal administration [110]. Oral administration of 17β-estradiol is not suitable, being more than 95% of the dose converted to metabolites before to reach the blood. Nasal administration of water soluble 17β-estradiol (**47**, **48**) allowed to obtain high levels of estradiol in the CSF [110]. Actually, a recent study in rat confirmed the existence of a direct transport route from the nasal cavity into the CSF for estradiol [111]. In this report, the *C*_max_ for estradiol in CSF was observed to be two-fold higher than that after intravenous administration, showing that after nasal delivery a large fraction of the estradiol dose reaches the CSF instead of the systemic circulation. To avoid the unwanted “feminizing” effects of the peripheric estrogen burden, the report of Prokai *et al*. [112] indicate a ‘‘chemical shield’’ erected by estrogens to protect neurons against the most harmful reactive oxygen species, the hydroxyl radical (•OH), involving a perpetuated antioxidant cycle through nonphenolic quinols as key intermediates, without affinity to the estrogen receptors and estrogenic activity (i.e., they are nonfeminizing). Quinols are rapidly converted back to the parent estrogens via an enzyme-catalyzed reduction by using NAD(P)H as a coenzyme (reductant) and, unlike redox cycling of catechol estrogens, without the production of reactive oxygen species. Due to this process, throughout neuroprotection against oxidative stress quinols act essentially as prodrugs for the active hormone [112].

Another example is the *n*-butyl ester of nipecotic acid, which was found to be deliverable into the brain. Total brain exposure to nipecotic acid was not found to be significantly different after nasal and intravenous administration of the prodrug. Rat brain disposition studies showed strong evidence that nipecotic acid formed *in vivo* from the ester may undergo tissue trapping, i.e. that the ester hydrolysis was the rate limiting to nipecotic acid brain accumulation [113].

## Conclusions

Developing prodrugs sensitive to brain-selective enzymatic cleavage by means of both CDS and lipidization procedures, although known since long time, is still one of the most promising strategies to deliver drugs in a sustained and specific manner to the CNS. Unfortunately, this result is still rather difficult to achieve because enzymatic activity in plasma often exceeds that in brain tissue, resulting in premature bioconversion of the prodrug. The needs to be met are to assure enzymatic stability to early degradation and exploitation of the “lock-in” mechanism to provide targeting. To this aim, new opportunities are represented by transporter gene cloning and *in vitro* uptake studies, which may be useful for design and synthesis of prodrugs selectively transportable by the CMTs expressed on brain barriers. Furthermore, an interesting strategy is suggested by the discovery of similarities between the type of carriers expressed in brain barriers and in nasal mucosa. Therefore, should this interesting hypothesis be confirmed, the best prodrug structures developed for crossing the blood-brain barrier could use the nasal pathway for more direct delivery into the CNS.
